# Illness Perceptions and Depression Are Associated with Health-Related Quality of Life in Youth with Inflammatory Bowel Disease

**DOI:** 10.1007/s12529-019-09791-6

**Published:** 2019-06-10

**Authors:** Luuk Stapersma, Gertrude van den Brink, Jan van der Ende, Alexander G. Bodelier, Herbert M. van Wering, Pamela C. W. M. Hurkmans, M. Luisa Mearin, Andrea E. van der Meulen–de Jong, Johanna C. Escher, Elisabeth M. W. J. Utens

**Affiliations:** 1 Department of Child and Adolescent Psychiatry/Psychology, Erasmus MC-Sophia Children’s Hospital, Wytemaweg 8, 3015 CN Rotterdam, The Netherlands; 2 Department of Pediatric Gastroenterology, Erasmus MC-Sophia Children’s Hospital, Rotterdam, The Netherlands; 3 Department of Gastroenterology, Amphia Hospital, Breda, The Netherlands; 4 Department of Pediatrics, Amphia Hospital, Breda, The Netherlands; 5 Department of Pediatrics, Leiden University Medical Center, Leiden, The Netherlands; 6 Department of Gastroenterology, Leiden University Medical Center, Leiden, The Netherlands; 7 Research Institute of Child Development and Education, University of Amsterdam, Amsterdam, The Netherlands; 8 Academic Center for Child Psychiatry the Bascule/Department of Child and Adolescent Psychiatry, Academic Medical Center, Amsterdam, The Netherlands

**Keywords:** Inflammatory bowel disease, Youth, Anxiety, Depression, Health-related quality of life

## Abstract

**Background:**

In youth with inflammatory bowel disease (IBD), health-related quality of life (HRQOL) has been shown to be affected by individual disease factors and specific psychological factors. The innovative aim of this study is to examine the *combined* impact of psychological factors (illness perceptions, cognitive coping, anxiety, and depression) on HRQOL, over and above the associations of demographic and disease factors with HRQOL in youth with IBD.

**Method:**

Data on clinical disease activity, illness perceptions, cognitive coping, anxiety, depression, and HRQOL were prospectively collected in 262 consecutive youth (age 10–20, 46.6% male) with confirmed IBD. Multiple linear regression analyses tested the associations of demographic, disease, and psychological variables with HRQOL in separate groups for Crohn’s disease (CD; *N* = 147) and ulcerative colitis and IBD unclassified (UC/IBD-U; *N* = 115), using age-specific validated instruments.

**Results:**

In both disease groups, more negative illness perceptions (*ß* = − .412; *ß* = − .438, *p* < .001) and more depression (*ß* = − .454; *ß* = − .279, *p* < .001) were related to lower HRQOL. In the UC/IBD-U group, more anxiety was related to lower HRQOL (*ß* = − .201, *p* = .001). The model with the psychological variables explained a large and significant amount of variance in both groups: 74% and 83%, respectively (*p* < .001).

**Conclusion:**

In 10–20-year-old IBD patients, negative illness perceptions and depression were significantly and more strongly associated with lower HRQOL than demographic and disease factors. Thus, it is important to integrate psychological factors in the treatment for IBD patients. To improve HRQOL in young IBD patients, psychological interventions should be targeted at negative illness perceptions and depression.

## Introduction

Inflammatory bowel disease (IBD) is a disabling chronic gastrointestinal condition, with two predominant subtypes: Crohn’s disease (CD) and ulcerative colitis (UC). In up to 25% of patients, IBD starts in late childhood or adolescence [1–3]. The designation IBD unclassified (IBD-U) is used for patients in which it is not (yet) possible to make a distinction between CD and UC. IBD is characterized by periods of clinical disease activity and remission and presents with symptoms such as abdominal pain, bloody diarrhea, fatigue, and weight loss [4]. In adolescence, growth failure and delayed pubertal development is common, specifically in Crohn’s disease. The adolescent life phase is characterized by development on several domains (biological, psychological, social, cognitive, academic). Having a chronic disease such as IBD can affect all these domains, for example, not only becoming more independent from parents, developing long-term friendships, starting secondary education, forming an own identity, but also experimenting with alcohol and drugs, seeking and finding a (side) job, and having romantic relationships. The teenage years are a crucial period of transition from childhood to young adulthood [5]. IBD and its medical treatment may severely impact psychosocial functioning: health-related quality of life (HRQOL) in children and adolescents (further referred to as youth) with IBD is significantly lower than in healthy peers [6, 7]. Furthermore, high prevalence rates varying from 20 to 50% for anxiety and depression are found in these patients [8–11]. A recent meta-analysis in children and adolescents showed pooled prevalence rates for anxiety and depressive symptoms of 15% and for anxiety and depressive disorders of 3–4% [12].

Other psychological factors are also important to consider in patients with IBD, such as illness perceptions and coping, because these have been shown to be related to psychosocial outcomes (such as HRQOL, general functioning or adjustment to IBD). Illness perceptions refer to the cognitive and emotional representations a patient forms about his or her disease [13]. These representations cover several dimensions, i.e., consequences (the expected effects of the disease), timeline (expectations about the duration of the disease), cause (thoughts about the cause of the disease), controllability (the extent to which the individual believes he or she can control the disease with or without treatment), identity (how the individual describes the symptoms and perceives as part of the disease), concern (worries about the disease), and emotions (the emotional response to the disease) [13, 14]. Coping refers to intentional efforts to regulate negative emotions in response to harm, threat, or challenges [15, 16] in this study dealing with IBD. Coping encompasses both cognitive and behavioral regulation. Cognitive coping is implicated in the etiology and the maintenance of anxiety and depression [17, 18] and is therefore important as well in studying youth with IBD.

The common sense model (CSM) is a model to describe the relationships between disease characteristics, illness perceptions, coping, and anxiety, depression, and HRQOL, originally developed by Leventhal and Diefenbach [13]. In this model, illness characteristics (such as clinical disease activity) lead to certain thoughts about the illness, the so-called illness perceptions of a patient. These illness perceptions influence the type of coping the patient uses to deal with his/her symptoms. Together, these factors lead to positive or negative illness outcomes, for example, anxiety, depression, or HRQOL. In patients with IBD, several relationships have been found between these variables, mostly in adults. Below it will be explicitly mentioned if studies were conducted in youth with IBD. For example, more clinical disease activity has been found to be associated with more anxiety and depression separately [19, 20]. Previous studies have also demonstrated a relationship between clinical disease activity and HRQOL, with a mediating role for anxiety and depressive symptoms [19, 21]. Furthermore, negative illness perceptions are associated with lower HRQOL in adults with IBD [22] and also with more psychological problems in youth with IBD [23]. Coping was associated with anxiety and depression [24] or adjustment to IBD [25] in adults and found as predictor of depression in youth with IBD as well [26].

Unfortunately, very little is known on how all the factors described above together affect health outcomes, more specifically HRQOL in young IBD patients. Only a few studies examined several psychological factors simultaneously. In adults, illness perceptions [27] and coping [24] have been reported to impact the relationship between clinical disease activity on HRQOL. Recently, Van Tilburg et al. [28] showed in adolescents with IBD that patient-reported disability (as outcome) was associated not only with clinical disease activity but also with a combined latent construct ‘psychological factors’ (including coping, pain beliefs, anxiety, and depression). However, they did not control for demographic factors (gender, age, socioeconomic status) and did not include other disease factors, such as disease type and disease duration. In addition, there is some evidence that these disease factors are associated with HRQOL [20, 29, 30] and with anxiety and/or depression as well [23, 31]. Moreover, because the authors used a combined psychological construct, their findings provide no insight on which psychological factors in particular psychological interventions for youth with IBD should focus.

The complex interplay between clinical disease activity, illness perceptions, coping, anxiety, and depression makes it challenging to attune both the medical and psychological treatment to the individual needs of IBD patients to improve their HRQOL. The surplus value of the present study in a cohort of youth with IBD is that it aims to clarify the association of a combination of psychological factors (illness perceptions, cognitive coping, anxiety, and depression) with HRQOL, over and above demographic and disease factors. By selecting the 10–20-year age range, we cover the period of transition from childhood to young adulthood. More specific insight on how psychological factors are associated with HRQOL in these vulnerable youth can offer guidance on which factors psychological interventions should focus. Ultimately, with tailored psychological interventions, the course of the IBD and of possible psychological problems may be positively affected. We hypothesize that clinical disease activity is negatively associated with HRQOL. Furthermore, we hypothesize that psychological factors (i.e., illness perceptions, cognitive coping, anxiety, and depression, when tested simultaneously) are associated with HRQOL, even after controlling for clinical disease activity and other demographic and disease factors.

## Materials and Methods

### Design

The present cross-sectional cohort study is based on a large patient sample (*N* = 374), completing the baseline assessment of a multicenter randomized controlled trial (RCT), investigating a disease-specific cognitive behavioral therapy in youth with IBD and symptoms of anxiety and/or depression (trial registration number: NCT02265588, see also Van den Brink and Stapersma et al. [32]). In the current study, only data from patients aged 10–20 years were used (*N* = 262).

*Inclusion criteria* were as follows: (1) age 10 to 20 years and (2) diagnosis of IBD, according to the consensus criteria [33] [34, 35].

*Exclusion criteria* were as follows: (1) intellectual disability; (2) current treatment for mental health problems (pharmacological and/or psychological); (3) insufficient mastery of the Dutch language; (4) a diagnosis of selective mutism, bipolar disorder, schizophrenia, autism spectrum disorder, obsessive-compulsive disorder, posttraumatic or acute stress-disorder, or substance use disorder; (5) cognitive behavioral therapy in the past year (at least eight sessions); and (6) participation in another intervention study.

### Procedure

Consecutive patients and their parents were recruited between October 2014 and September 2016 from the outpatient clinic in two academic hospitals and four community hospitals in the Southwest region of the Netherlands. Patient information was given, and written informed consent was requested in all patients and, if applicable, their parents or caregivers. Patients (and parents), who consented to participate, received an e-mail with a link to online questionnaires. Clinical disease activity was scored by the (pediatric) gastroenterologist around the time of inclusion (i.e., within approximately a month around the time of inclusion, median = 3.42 weeks).

### Measures

#### Control Variables

*Gender*, *age*, *disease type*, and *disease duration* of the patients were derived from their medical record. *Socioeconomic status* (SES) was determined using the occupational level from the parents or, if they lived on their own, patients themselves. Using the standard coding system of Statistics Netherlands ([36]), occupations were categorized in low, middle, and high. For gender and SES, dummy variables were created to use in the analyses.

*Clinical disease activity* was assessed by two validated clinical disease activity instruments. For CD, the short Pediatric Crohn’s Disease Activity Index (sPCDAI) and, for UC, the Pediatric Ulcerative Colitis Activity Index (PUCAI) were used. The sPCDAI comprises six items on medical history (abdominal pain, stools), well-being, physical examination (abdomen), weight, and extra-intestinal manifestations [37]. Scores range from 0 to 90 points [38]. The PUCAI comprises six items on abdominal pain, rectal bleeding, stool frequency and consistency, and activity level. Scores range from 0 to 85.

#### Psychological Factors

*Illness perceptions* were assessed by the Brief Illness Perceptions Questionnaire (B-IPQ; [14, 39]). This 9-item self-report questionnaire assesses cognitive and emotional representations of illness, covering eight dimensions: consequences, timeline, personal control, treatment control, identity, concern, emotions, and understanding. All dimensions are scored on an 11-point Likert-scale (0: not at all–10: very much/severely). A higher score represents more negative illness perceptions. Good test–retest reliability and concurrent validity have been found [14], and the B-IPQ has been used before in adolescents with IBD [40]. Internal consistency (Cronbach’s alpha) for the current sample was .81 in the CD group and .81 in the UC/IBD-U group.

*Cognitive coping* was measured with the Cognitive Emotion Regulation Questionnaire (CERQ). This self-report scale consists of 36 items, scored 1 to 5 points, with nine subscales (e.g., self-blame, acceptance, putting into perspective, positive refocusing, positive reappraisal, and catastrophizing). These scales are divided into two domains: adaptive cognitive coping (e.g., positive reappraisal, putting in perspective) and maladaptive cognitive coping (e.g., self-blame, catastrophizing). A higher score indicates more use of a particular coping style. Good reliability and construct validity have been found [41]. Both adaptive coping and maladaptive coping were used as variable in the analyses. For adaptive cognitive coping, internal consistency was .90 in the CD group and .93 in the UC/IBD-U group. For maladaptive cognitive coping, internal consistency was .88 in the CD group and .90 in the UC/IBD-U group.

*Anxiety* was assessed using the 69-item self-report questionnaire Screen for Child Anxiety Related Disorders (SCARED). The SCARED contains five subscales: general anxiety disorder, separation anxiety disorder, specific phobia, panic disorder, and social phobia, rated on a 3-point scale (0–2: total score 0–138). Satisfactory reliability and validity have been reported [42]. The cutoffs for elevated anxiety were total score ≥ 26 for boys, ≥ 30 for girls, or subscale score ≥ 8 [43]. These were only used to decide whether patients had elevated anxiety, i.e., could be included in the RCT. Internal consistency for the current sample was .95 in the CD group and .94 in the UC/IBD-U group.

*Depression* was assessed using the Child Depression Inventory (CDI, for ages 10–17) and the Beck Depression Inventory, second version (BDI-II, for ages 18–20). The CDI is a 27-item self-report scale (0–2, total score 0–54). Good reliability and validity have been established. A CDI score of 13 or higher reflected elevated depression [44]. The BDI-II is a 21-item self-report scale (0–3, total score 0–63). It has excellent reliability and good to excellent validity. A BDI-II score of 14 or higher reflected elevated symptoms of depression [45]. The cutoffs for the CDI and BDI-II were only used to decide whether patients had elevated depression, i.e., could be included in the RCT. For the CDI, internal consistency was .85 in the CD group and .86 in the UC/IBD-U group. For the BDI-II, internal consistency was .91 in the CD group and .84 in the UC/IBD-U group. To be able to combine patients of all ages within the disease groups, depression scores were created a *Z*-score for depression using either the CDI or the BDI-II (depending on age).

*Health-related quality of life* was assessed by the IBD-specific self-report IMPACT-III, which covers six domains: IBD-related symptoms, systemic symptoms, emotional functioning, social functioning, treatment related concerns, and body image [46]. The 35 items are scored (1–5; total score 35–175). Good psychometric properties have been found [47]. The total score was used, and a higher total score indicates better HRQOL. Although the IMPACT-III originally was designed for youth up to 18 years, we also used it for the patients of 19 and 20 years. This allowed us to combine all patients in to one group for each disease type. This was substantiated by excellent internal consistency in both disease groups: .93 in the CD group and .94 in the UC/IBD-U group.

### Statistical Analyses

To test whether the associations of demographic, disease and psychological factors with HRQOL are different for CD than for UC/IBD-U, multiple linear regression analyses were performed for the two disease groups separately: CD (*N* = 147) and UC/IBD-U (*N* = 115). UC and IBD-U were combined, since the group with IBD-U patients was quite small (*N* = 18), IBD-U often resembles UC more than CD [48], and IBD-U has often a similar treatment approach as UC [49]. All variables were continuous, except for gender and SES. For these variables, dummy variables were included in the analyses. The abovementioned cutoffs for the SCARED, CDI, and BDI-II were only used to determine the proportion of patients with elevated anxiety or depressive symptoms. For the remaining questionnaires, no cutoffs were used.

The multiple linear regression analyses were run with two blocks/models, using HRQOL as outcome. In the first block, the demographic and disease variables (gender, age, disease duration, SES, and clinical disease activity) were entered simultaneously in the first regression model. In the second block, the psychological factors (illness perceptions, cognitive coping, anxiety, and depression) were added simultaneously to the variables entered in the first block. To account for missing values, multiple imputation with chained equations was applied using SPSS (*m* = 15 for approximately 15% of missing data; [50]). As a sensitivity analysis, we also performed a complete case analysis (*N* = 116; *N* = 104 for the CD and UC/IBD-U groups, respectively) to see whether the multiple imputations had an effect on the results. A *p* value of < .05 was considered significant. SPSS Version 24 was used for the analyses (IBM Corp. Released 2016. IBM SPSS Statistics for Windows, Version 24.0. Armonk, NY: IBM Corp). A statistician (JE) supported and advised in analyzing and interpreting the data and results.

### Ethical Considerations

This study was performed conform the Declaration of Helsinki and approved by the Institutional Review Board of the Erasmus MC and of each participating center.

## Results

### Patient Characteristics

In total, 552 patients (aged 10–25 years) were invited for a randomized controlled trial (RCT) and 382 agreed to participate (response rate = 69%). Eight patients had incomplete data. From the final 374 youth, 262 were aged 10–20 years and were included in the current study. Demographic, disease, and psychological characteristics are presented in Table 1. In disease groups (CD and UC/IBD-U), the percentage of patients with active disease (mild-moderate-severe) was 31.3% and 30.4%, respectively. Overall, 50% of the patients had elevated anxiety, 17.9% had elevated depression, and 16.8% had both. For HRQOL, illness perceptions, and cognitive coping, no cutoffs are available; so means with ranges are provided in Table 1.

**Table 1:** Demographic and disease characteristics of total sample of IBD patients (10–20 years)

		Group		Total
		CD	UC/IBD-U	
*N*		147	115	262
Demographic characteristics				
Age (years), mean (SD)		17.19 (2.52)	16.15 (2.98)	16.74 (2.77)
Male (%)		50.3	41.7	46.6
SES (%)^a^	Low	17.7	13.0	15.6
	Middle	32.7	40.9	36.3
	High	38.1	43.5	40.5
Disease characteristics				
Disease type (%)	CD	100	0	56.1
	UC	0	84.3	37.0
	IBD-U	0	15.7	6.9
Age at diagnosis (years), mean (SD)		14.25 (3.02)	13.18 (3.97)	13.78 (3.50)
Disease duration (years), median (IQR)		2.26 (0.97–4.39)	1.90 (0.62–4.73)	2.03 (0.81–4.56)
Active disease (%)^b^		31.3	30.4	30.9
Psychological characteristics				
Elevated anxiety symptoms (%)^c^		49.7	50.4	50.0
Elevated depressive symptoms (%)^c^		15.6	20.9	17.9
Elevated anxiety and depression (%)^c^		14.3	20.0	16.8
HRQOL, mean (range)		143.36 (76–174)	141.91 (82–173)	142.72 (76–174)
Illness perceptions, mean (range)		34.75 (3–69)	36.69 (9–70)	35.63 (3–70)
Adaptive cognitive coping, mean (range)		57.67 (20–92)	55.72 (21–97)	56.78 (20–97)
Maladaptive cognitive coping, mean (range)		25.05 (16–59)	25.12 (16–56)	25.08 (16–59)

*SD* standard deviation, *SES* socioeconomic status, *CD* Crohn’s disease, *UC* ulcerative colitis, *IBD-U* inflammatory bowel disease unclassified, *IQR* interquartile range, *HRQOL* health-related quality of life, *sPCDAI* short Pediatric Crohn’s Disease Activity Index, *PUCAI* Pediatric Ulcerative Colitis Activity Index, *SCARED* Screen for Child Anxiety Related Emotional Disorders, *CDI* Child Depression Inventory, *BDI-II* Beck Depression Inventory, Second edition

^a^Not for all patients SES was available: CD *N* = 130, UC/IBD-U *N* = 112. Total group *N* = 242

^b^Scored above cutoff for active disease on sPCDAI (≥ 10 points) or PUCAI (≥ 10 points)

^c^Scored above cutoff on SCARED (anxiety), CDI (depression; 10–17 years) and/or BDI-II (depression 18–20 years)

### Influence of Demographic, Disease, and Psychological Variables on HRQOL; Results of Multiple Linear Regression Analyses per Disease Group

In Tables 2 and 3, the standardized estimates, their significance, and the proportion of explained variance for each regression model (model 1 with demographic and disease factors and model 2 with psychological factors added) in the two disease groups are provided. Results are presented from the analyses on the imputed datasets. Results from the complete case analyses were similar (data not shown).

**Table 2:** Influence of demographic, disease, and psychological variables on HRQOL; results of multiple linear regression analysis—CD group (*N* = 147)

		Model 1			Model 2		
		*ß*	SE *ß*	*p* value	*ß*	SE *ß*	*p* value
Block 1							
Gender	Male						
	Female	− .230	.072	.002*	− .055	.051	.283
Age		− .075	.077	.330	− .054	.056	.330
SES	Low						
	Middle	.029	.098	.768	.070	.069	.315
	High	.051	.097	.600	− .042	.067	.533
Disease duration		.123	.072	.086	.064	.049	.193
Clinical disease activity		− .482	.071	< .001*	− .170	.053	.001*
Block 2							
Illness perceptions					− .412	.063	< .001*
Adaptive cognitive coping					.012	0.49	.803
Maladaptive cognitive coping					.018	.058	.759
Anxiety					.019	.077	.801
Depression					− .454	.077	< .001*
*R*^2^ (CI), *p* value		.37 (.24–.49), *p* < .001*			.74 (.65–.80), *p* < .001*		

*SE* standard error, *SES* socioeconomic status, *CI* confidence interval

* significant with *p* < .05

**Table 3:** Influence of demographic, disease, and psychological variables on HRQOL; results of multiple linear regression analysis—UC/IBD-U group (*N* = 115)

		Model 1			Model 2		
		*ß*	SE *ß*	*p* value	*ß*	SE *ß*	*p* value
Block 1							
Gender	Male						
	Female	− .276	.081	.001*	− .101	.044	.022*
Age		− .298	.082	< .001*	− .193	.045	< .001*
SES	Low						
	Middle	− .130	.126	.302	− .033	.066	.619
	High	− .101	.126	.424	− .047	.067	.483
Disease duration		.201	.083	.015*	.087	.044	.045*
Clinical disease activity		− .289	.081	< .001*	− .066	.046	.156
Block 2							
Illness perceptions					− .438	.056	< .001*
Adaptive cognitive coping					.036	.046	.156
Maladaptive cognitive coping					− .029	.052	.584
Anxiety					− .201	.060	.001*
Depression					− .279	.061	< .001*
*R*^2^ (CI), *p* value		.32 (.18–.46), *p* < .001*			.83 (.76–.88), *p* < .001		

*SE* standard error, *SES* socioeconomic status, *CI* confidence interval

* significant with *p* < .05

As is seen in Table 2, in the CD group (*N* = 147), female gender and clinical disease activity were significantly associated with HRQOL in the first model, explaining 37% of the variance in HRQOL. After adding the psychological factors, clinical disease activity (*ß* = − .170, *p* = .001), more negative illness perceptions (*ß* = − .412, *p* < .001), and more depressive symptoms (*ß* = − .454, *p* < .001) were associated with lower HRQOL. The second model explained 74% of the variance in HRQOL, with a significant change in explained variance (*R*^2^ change = 37%, *p* < .001).

In the UC/IBD-U group (*N* = 115), female gender, age, disease duration, and clinical disease activity were significantly associated with HRQOL in the first model, explaining 32% of the variance in HRQOL. After adding the psychological factors, female gender (*ß* = − .101, *p* = .022), lower age (*ß* = − .193, *p* < .001), shorter disease duration (*ß* = .087, *p* = .045), more negative illness perceptions (*ß* = − .438, *p* < .001), more anxiety symptoms (*ß* = − .201, *p* = .001), and more depressive symptoms (*ß* = − .279, *p* < .001) were associated with lower HRQOL. The second model explained 83% of the variance, with a significant change in explained variance (*R*^2^ change = 51%, *p* < .001).

## Discussion

This study examined the influence of psychological factors on HRQOL over and above the influence of demographic and disease factors in youth with IBD and analyzed the results separately for CD and UC/IBD-U. Partly in line with our first hypothesis, in the first model, without the psychological factors included, female gender and clinical disease activity were significantly associated with HRQOL, as were age and disease duration only in the UC/IBD-U group. However, when adding a combination of all psychological factors simultaneously in the second model, the influence of demographic and disease factors was reduced. Subsequently, illness perceptions and depression were associated with HRQOL in youth with IBD, even when controlling for demographic and disease factors. More negative illness perceptions and more depression were associated with a lower HRQOL, in both the CD group and the UC/IBD-U group. A difference between the disease groups was that, in the UC/IBD-U group, anxiety was associated with HRQOL as well. Most importantly, adding the psychological factors resulted in a significant increase in the proportion of explained variance, from approximately 35% by the first model to 74–83% by the second model, in both groups. This high proportion of explained variance underlines the importance of psychological factors contributing to HRQOL in patients with IBD.

These results provide insight in which psychological factors play a role in youth with IBD. Consistently in the two disease groups, negative illness perceptions and depression in particular prove their significant role, whereas cognitive coping was not associated with HRQOL. This was also found in previous studies, which reported that illness perceptions and depression were associated with disease outcomes [22, 28]. Therefore, we recommend to pay attention to these factors when treating patients. Our results suggest that in youth with UC/IBD-U, anxiety should be considered as well.

There are several explanations for only finding an association between anxiety and HRQOL in youth with UC/IBD-U. Firstly, the nonsignificant relationship between anxiety and HRQOL in youth with CD cannot be explained by a difference in the prevalence of elevated anxiety symptoms between the CD and UC/IBD-U groups (49.7% versus 50.4%). Secondly, one might postulate that anxiety is not strongly related to HRQOL in youth with IBD. Although we found a high prevalence of anxiety symptoms in the current sample (see Table 1 and [31]), the presence of anxiety symptoms as such may not have to impact the HRQOL of youth with CD. In children and adolescents, the available studies did not show evidence for differences between CD and UC [23], but Sarid et al. [51] showed worse psychosocial outcomes in patients with UC [27, 52]. Thirdly, anxiety and depression are highly comorbid, have overlapping symptoms, and anxiety is considered a precursor of depression [53, 54]. So anxiety may have played a role in preceding depressive symptoms in these patients. It is possible that anxiety and depression both explained variance in HRQOL, but that depression is more strongly related to HRQOL and therefore diminished the relationship between anxiety and HRQOL in the patients with CD. More research is needed to unravel the interplay between anxiety and depression in youth with IBD. In their benchmark review, Cummings et al. [55] describe several pathways for the anxiety and depression comorbidity in children and adolescents. They also stress the importance of studying specific anxiety disorders for their comorbidity with depression. In IBD, very few studies tested specific anxiety problems (e.g., [11]). As a result, to our knowledge, there are no studies that investigated how specific anxiety problems are related to depressive symptoms in patients with IBD. Fourthly, in adults, several studies reported on the relationship between anxiety and HRQOL in both patients with CD and UC [56, 57]. Anxiety might be more impairing for adults with IBD than for youth with IBD, since adults may have to deal with more disease-related anxieties and worries concerning their daily and social functioning (impact of IBD on employment, career perspectives, income, finding a sexual partner, starting a family, etc.). Lastly, anxiety symptoms may be IBD-specific, i.e., anxiety or worries surrounding their IBD symptoms (e.g., bloody stools, the necessity of a stoma or surgery). These worries are often exorbitant to the actual context but can have a negative impact [58, 59]. More specifically, higher IBD-specific anxiety was associated with lower HRQOL in youth with both CD and UC [58]. However, we are not aware of studies that examined differences between CD and UC with respect to IBD-specific anxiety in youth. Perhaps, youth with UC/IBD-U experience different IBD-specific worries than youth with CD, for example, since youth with UC/IBD-U more often have alarming bloody stools than youth with CD.

Although the CSM postulates that coping is an important factor, in our study, cognitive coping was not significantly related to HRQOL, when simultaneously added to the model with the other psychological factors. Cognitive coping may not be related to HRQOL in IBD patients, as was also found in earlier studies examining individual psychological factors [27, 52]. This is in contrast with the results of a review including a wide range of illnesses in adults, which found that coping was a stronger predictor for health outcomes than illness perceptions [60]. Perhaps, coping plays a different role in IBD than in other illnesses. On the other hand, the type of coping may be of importance, since we only tested cognitive coping styles (and not for example behavioral). However, the results of two adult IBD studies including problem-focused and emotion-focused coping are mixed [61, 62] and therefore do not support this explanation completely.

Comparing the results between patients with CD and UC/IBD-U showed differences in the second model: a significant association of clinical disease activity with HRQOL in the CD group and a significant association of gender, age, and disease duration with HRQOL in de UC/IBD-U group. Most likely, these differences cannot be explained by differences between the two groups, since the groups were similar with respect to the percentages of active disease, males versus females and the disease duration (see Table 1). Only in the CD group, clinical disease activity was associated with HRQOL, even after adding the psychological factors to the model. To our knowledge, in youth, no studies have specifically examined differences between CD and UC with respect to the relationship between clinical disease activity and HRQOL. Patients with CD have a more heterogeneous clinical presentation and are affected by growth failure, more often than patients with UC and IBD-U [1]. The heterogeneous clinical presentation and growth failure can lead to a lower HRQOL. A recent review of Knowles et al. [63] showed that HRQOL was significantly lower for patients with active disease, although no information was provided about differences between CD and UC/IBD-U. In children and adolescents with IBD (both CD and UC), some studies have shown that clinical disease activity remained associated with HRQOL, even when anxiety/depression [19, 21, 64] and parental stress [64, 65] were included as mediators. It was therefore not tested, as in our study, what the influence is of demographic, disease, and psychological factors on the relationship between clinical disease activity and HRQOL. It seems that the relationship between clinical disease activity and HRQOL is not a direct relationship as such.

Only in the UC/IBD-U group, gender, age, and disease duration were significantly associated with HRQOL, even after adding the psychological factors to the model. For gender, previous studies in youth with IBD did not find an association with HRQOL [66–68]. These studies all included both CD and UC patients, but the majority of youth had CD (> 70%), which may have masked the association between gender and HRQOL in the UC patients. However, it remains unclear what role gender has in affecting HRQOL, especially since gender is associated with more anxiety and depressive symptoms in general [69], as well as in our own cohort [31]. Anxiety and depressive symptoms are known to affect HRQOL in youth with IBD [21, 64]. For age, our results indicated that older age was associated with lower HRQOL in youth with UC/IBD-U. This is accordance with Otley et al. [68], who also reported that older age was associated with lower HRQOL in the first year after diagnosis of IBD. However, in their sample, a large majority of youth was diagnosed with CD (77%). Other studies did not find association between age and HRQOL [30, 70] or the reversed association (lower HRQOL in younger patients; [20]). These mixed findings were confirmed in a review on predictors of HRQOL in youth with IBD [6]. Finally, in our study, a shorter disease duration was associated with a lower HRQOL in youth with UC/IBD-U. Previous studies have suggested that it seems that disease duration is not associated with HRQOL in general, but only within the first months after diagnosis (of both CD and UC/IBD-U) [30, 68]. However, these studies included mainly CD patients (77% and 100%, respectively) [30, 68]. In our sample, only 20% had a disease duration of 6 months or shorter. Therefore, our results suggest that for youth with CD in the first 6 months after the diagnosis, disease duration is not associated with HRQOL. For UC and IBD-U, this relationship is unclear less clear defined. Although these differences between the disease groups are important to notice, the most important finding remains that, overall, in both disease groups, illness perceptions and depression were significantly associated with HRQOL.

### Strengths

Our sample (*N* = 262) is one of the largest European samples and innovative in studying the influence of both disease, demographic, and psychological factors in youth with IBD. Our large sample covers a broad age range, using internationally validated and age-attuned instruments. This age range is an important life phase, as several biological and psychosocial changes take place, and a chronic disease such as IBD can have negative consequences for the transition to adulthood. In addition, our sample was derived from 6 centers (both urban and rural areas), making generalization of our findings stronger. In addition, and contrary to other studies that only included either anxiety or depression (e.g., [8, 11]), we assessed both anxiety and depression, as this had implications for subsequent psychological treatment. Most importantly, whereas previous studies mostly examined individual relationships between disease factors, psychological factors and HRQOL, we aimed to test the influence of disease, demographic, and psychological factors simultaneously. An advantage of this approach is that the current study took into account the interrelationships between all the factors in their associations with HRQOL.

### Limitations

The number of patients with active disease (mostly mild clinical disease activity) was low (25%), although this number is often found in population-based cohorts of patients with IBD. It may be that the associations between the psychological variables and HRQOL are not the same for patients that have more active disease. Nevertheless, studies with a higher proportion of patients with active disease reported similar results [6, 21, 64, 65]. However, despite these findings, it is still possible that for patients with moderate to severe clinical disease activity, the relationships between illness perceptions, depression, and HRQOL are different. Since evidence has been found for a negative impact of clinical disease activity on anxiety, depression, and/or HRQOL (e.g., [11, 23]), even stronger relationships may be found in patient populations with more active disease. Another limitation is that our data were cross-sectional and conclusions on causal relationships cannot be drawn. Longitudinal studies are needed to examine causal relationships over time. Until now, only few studies have been conducted that were able to draw conclusion on causal relationships. For example, a recent study in adults with IBD found evidence for a bidirectional and causal relationship between disease activity and anxiety/depression [71]. However, such studies have not been conducted investigating HRQOL. A last limitation is that we had a response rate of approximately 70%, which can have caused bias, for example, if patients with a lower HRQOL were more inclined to participate than those with higher HRQOL. However, we were not able to compare the HRQOL of responders and nonresponders.

### Clinical Implications

These results stress the importance of psychological factors for HRQOL in youth with IBD, over and above demographic and disease variables. In our study sample, 75% of the patients were in clinical remission. Therefore, treating (pediatric) gastroenterologists should pay attention to these psychological factors, in all patients and not only in patients with active disease. We recommend screening for negative illness perceptions and internalizing problems. This can be done either during a medical visit or using short (online) questionnaires prior to the medical visit. Our results also have implications for psychological treatment of these patients: interventions for improving HRQOL should focus on negative illness perceptions and depression and also on anxiety for youth with UC/IBD-U. For example, cognitive behavioral therapy (CBT) has been proven effective in using techniques to restructure thoughts, such as negative illness perceptions [72]. Importantly, at the beginning of a psychological intervention disproportionate, unrealistic or incorrect thoughts and ideas should be identified. At this phase, it is important to determine whether a patient has disproportionate or incorrect negative illness perceptions. These can then be crucial when practicing cognitive and behavioral techniques. Naturally, the techniques of CBT can be used to improve depression and anxiety as well [73, 74].

In conclusion, our study found that negative illness perceptions and depression are negatively associated with HRQOL in youth with IBD, even after controlling for several demographic and disease factors, with also other psychological factors (i.e., coping, anxiety) taken into account. These factors seriously influence HRQOL, even in our cohort with low clinical disease activity, and should be considered by the medical team. Our results indicate that, irrespective of the clinical disease activity, psychological treatment should focus on the way these young IBD patients perceive their disease and on their depressive symptoms. For youth with UC and IBD-U, anxiety and worries should receive attention as well.
